# Effect of Intercalation Structure of Organo-Modified Montmorillonite/Polylactic Acid on Wheat Straw Fiber/Polylactic Acid Composites

**DOI:** 10.3390/polym10080896

**Published:** 2018-08-09

**Authors:** Qiqi Fan, Guangping Han, Wanli Cheng, Huafeng Tian, Dong Wang, Lihui Xuan

**Affiliations:** 1 Key Laboratory of Bio-Based Material Science and Technology (Ministry of Education), Northeast Forestry University, Harbin 150040, China; fanqiqi@nefu.edu.cn (Q.F.); guangping.han@nefu.edu.cn (G.H.); zcwangd@hotmail.com (D.W.); leeh91@hotmail.com (L.X.); 2 Beijing Key Laboratory of Quality Evaluation Technology for Hygiene and Safety of Plastics, Beijing Technology and Business University, Beijing 100048, China

**Keywords:** organo-modified montmorillonite, two-step treatment, wheat straw fiber/polylactic acid composites, dopamine, mechanical properties

## Abstract

In this work, an easy way to prepare the polylactic acid (PLA)/wheat straw fiber (WSF) composite was proposed. The method involved uses either the dopamine-treated WSF or the two-step montmorillonite (MMT)-modified WSF as the filler material. In order to achieve the dispersibility and exfoliation of MMT, it was modified by 12-aminododecanoic acid using a two-step route. X-ray diffraction (XRD) and scanning electron microscopy (SEM) were performed to characterize the modified MMT and the coated WSF. As for the properties of PLA/WSF composites, some thermal (using Fourier transform infrared spectroscopy (FT-IR) and thermogravimetric analysis) and mechanical properties (flexural, tensile, and impact) were analyzed. The results showed that the dopamine was successfully coated onto the WSF. Furthermore, Na-MMT was successfully transformed to organo-montmorillonite (OMMT) and formed an exfoliated structure. In addition, a better dispersion of MMT was obtained using the two-step treatment. The interlayer spacing of modified MMT was 4.06 nm, which was 123% higher than that of the unmodified MMT. Additionally, FT-IR analysis suggested that OMMT diffused into the PLA matrix. The thermogravimetric analysis (TGA) showed that a higher thermal stability of PLA/WSF composites was obtained for the modified MMT and dopamine. The results also showed that both the dopamine treated WSF and the two-step-treated MMT exhibited a positive influence on the mechanical properties of PLA/WSF composites, especially on the tensile strength, which increased by 367% compared to the unmodified precursors. This route offers researchers a potential scheme to improve the thermal and mechanical properties of PLA/WSF composites in a low-cost way.

## 1. Introduction

Due to increasing environmental problems and restricted availability of petrochemical resources, biopolymers have become popular among researchers in recent years. PLA, is a biopolymer, and is a promising material for various end-use applications. Currently, there is an increasing interest in using PLA for disposable and degradable plastic products [1,2]. However, some disadvantages such as high cost, flexural properties, heat distortion temperature (HDT), gas permeability, impact factor, melt viscosity for further processing hinder the application of PLA for manufacturing end-use products. Numerous studies have focused on improving the properties as well as decreasing the price of PLA by mixing it with some natural fiber materials [3,4,5,6], synthetic monomers and polymers, such as polyethylene glycol (PEG) [7], glycerol maleic anhydride (MA) [8] and many kinds of inorganic fillers [9,10,11,12].

WSF is an agricultural residue and is an abundant natural material. The burning off of wheat straw and other agricultural residues results in not only a series of environmental problems but also causes the waste of precious resources. Therefore, the use of a waste biomass resource to produce useful end products has attracted considerable attention in recent years. Recently, many studies have been conducted on composite panels marked by various agricultural residues and plastic [13,14,15]. The most commonly investigated mechanical properties of agricultural fiber plastic composites (AFPC) are comparable to wood-plastic composites (WPC), and include tensile, flexural, and impact properties. Similar to WPC, AFPC can be extensively employed in various industries, such as furniture, packaging, transportation and decorative materials. However, hydrophilic agricultural fibers are incompatible with hydrophobic bioplastics, which results in poor interfacial adhesion between the fiber and the matrix. This in turn results in reduced mechanical properties of the resultant composite [16]. Therefore, it is necessary to improve the interfacial adhesion capacity between natural agricultural fibers and the matrix. Recently, polydopamine (PDA) has attracted significant attention in interface modification due to its capability to increase the biocompatibility of a surface. To the best of our knowledge, PDA coupled with WSF has not yet been reported for its potential to modify the interfaces favorably.

MMT is the most widely used clay and is another kind of filler reinforcement, which has attracted a great deal of interest in both the fiber products and the polymers. It is a layered silicate with the mean layer thickness of 0.96 nm. Chemically, MMT mainly consists of Al_2_O_3_·4SiO_2_·3H_2_O with two layers of tetrahedron of Si–O and one layer of octahedron with Al–O in between. When layered silicate is introduced into wood or polymer matrix, three types of structures are commonly obtained. These structures are: (1) a phase-separated structure; (2) an intercalated structure; and (3) an exfoliated or delaminated structure [17]. An exfoliated structure is always expected to form a true nanocomposite due to superior interfacial adhesion. Recently, many studies have focused on improving the interfacial adhesion between MMT and PLA. Wang et al. [18]. Used OMMT modified by didecyl dimethyl ammonium chloride to treat wood and found that the compression strength of the treated wood increased by 27% compared to the untreated control, and the decay resistance of the composite was also improved. However, the route, used by the authors [18,19], suffers from poor performance, which could be due to poor exfoliation by didecyl dimethyl ammonium chloride. Therefore, a new efficient route to improve the exfoliation of MMT needs to be developed. The proposed two-step route for improving the dispersibility and exfoliation of MMT would be a potential scheme, involving the exfoliation by 12-aminododecanoic acid and the coupling using various coupling agents.

As mentioned earlier, the aim of this study was to investigate the practical feasibility of WSF coupled with PDA and PLA intercalating OMMT to manufacture the PLA/WSF composites. In order to achieve the dispersibility and exfoliation of MMT, 12-aminododecanoic acid with a two-step route was used for modifying the MMT. In addition, XRD and SEM were performed to study the modified MMT and coated WSF. As for the properties of PLA/WSF composites, some thermal (using FT-IR and TGA) and mechanical properties (such as, flexural, tensile, and impact) were studied to verify the improvement achieved using the proposed modification process.

## 2. Materials and Methods 

### 2.1. Raw Materials and Preparation

PLA (AI-1001) (L content) was obtained from Nature Works (Blair, NE, USA). It had a density of around 1.25 g/cm^3^ and the melt flow index was about 10–20 g/10 min at 190 °C. The molecular weight (Mn) of PLA was about 80,000. Wheat stalk was collected from a farmland in Weifang, China. The wheat stalk was granulated to the length of 40–80 μm, after which, the granules were dried to a moisture content of approximately 3% prior to further use. Na-montmorillonite (Na–MMT) was purchased from Zhejiang Fenghong Clay Chemical Co., Ltd., Huzhou, China. The viscosity of 3% (*w*/*w*) Na–MMT in distilled water was 3000 MPa·s with the pH of 8–10. The mean interlayer distance of Na–MMT was 1.459 nm with the particle size of 76 μm. The cation exchange capacity of Na–MMT was 90 mmol/100 g. The modifier used in this study was 12-aminododecanoic acid (ALA; 98%), which was purchased from Huaxia Reagent Co., Ltd., (Chengdu, China). In addition, aminopropyltriethoxysilane (KH550; 99%), dopamine hydrochloride (98%), polyethylene glycol (average *M*n of 1000 g/mol), zinc oxide (ZnO), industrial paraffin, distilled water, and ethanol were purchased from Aladdin Reagent Co., Ltd., (Shanghai, China).

### 2.2. Modification of Montmorillonite

Although functionalized MMT nanoparticles can be obtained from many commercial suppliers, there are certain issues, which affect the control quality and the stability of the filler. Therefore, the MMT nanoparticles used in this study were functionalized in the laboratory. ALA was used to exchange the Na cation of Na-MMT with the long organic cation of ALA to form OMMT as well as to enlarge the interlayer distance of MMT. The concentration of ALA was calculated according to the concentration of Na-MMT at the ratio of cation exchange capacity of Na-MMT of 0.7:1. The ALA-modified MMT was heated at 80 °C in a water bath. The obtained product was ultrasonicated for 5 min and stored for 12 h. Before packing, the sample was dried at 60 °C for 4 h and then, ground.

### 2.3. Preparation of Wheat Straw Fiber

The WSF was dried in an oven at 103 ± 2 °C until the moisture content was reduced to around 1–2%. A 2 g/L dopamine aminomethane solution (pH of 8.5; 0.1 mol/L) was prepared [20,21,22,23,24,25]. The dopamine aminomethane solution was added to WSF under rigorous washing for 15 min. Subsequently, the modified WSF was dried at 103 ± 2 °C to reach the same moisture content with unmodified WSF.

### 2.4. Preparation of Composites

A comprehensive analysis of the relevant formulations of biomass composites was conducted. Based upon the analysis, the design for various formulations is presented in Table 1.

### 2.5. Material Characterization

#### 2.5.1. Characterization Using SEM

The powder of five different clays and the internal surfaces of the samples were examined using a JSM-7500F scanning electron microscope (FEI Company, Eindhoven, The Netherlands) with the acceleration voltage of 10 kV. The samples were sputter-coated with gold before observation.

#### 2.5.2. XRD Analysis

Wide-angle X-ray diffraction (WXRD) patterns of the MMT samples were obtained before and after the modification using a D/MAX 2200 X-ray diffractometer (Rigaku Co., Ltd., Sendagaya, Japan). The X-ray beam was Cu-Kα (with the wavelength (*λ*) of 0.1540 nm) radiation, and the equipment was operated at 40 kV and 30 mA. The scanning rate was 2°/s, while the 2*θ* ranged from 10° to 80°. The interlayer spacing of MMT can be calculated using the Bragg equation:

(1) $$d=\frac{n\lambda }{2\sin\theta }$$

where *d* refers to the interlayer distance; *n* refers to the integer wavelength number (*n* = 1); *λ* refers to the X-ray wavelength, and θ refers to the maximum diffraction angle.

#### 2.5.3. FTIR Analysis

The treated and untreated WSF and PLA/WSF composites were ground and the FTIR absorption data were obtained using a Nicolet 6700 FTIR spectrometer (Thermo Fisher Scientific Pty., Ltd., Agawam, MA, USA) with an attenuated total reflection (ATR) unit (universal ATR diamond Zn/Se) at a resolution of 4 cm^−1^ for 32 scans in the spectral range of 600–4000 cm^−1^.

#### 2.5.4. Thermogravimetric Analysis

Thermal properties of the untreated and treated PLA/WSF composites were measured using TGA 309F3 thermal analyzer (TA Instruments, New Castle, DE, USA) at a heating rate of 10 °C /min from room temperature to 600 °C under a nitrogen atmosphere.

#### 2.5.5. Mechanical Properties of the WSF/PLA Composites

The flexural tests were conducted according to the ASTM D-790-03 standard, which uses a support span of 64mm and involves a three-point bending test at a crosshead speed of 2 mm/min. The size of the samples was 80 mm × 13 mm × 4 mm (thickness). The tensile tests were carried out according to the ASTMD638-03 standard at a testing speed of 5 mm/min. The dimensions of the samples were 165 mm × 19 mm × 4 mm (thickness). All samples possessed a standard dumbbell-shape with a middle width of 13 mm. Five replicates were run for each formulation. All tests were performed on a universal testing machine (Shenzhen REGER Instrument Co., Ltd., Shenzhen, China). The impacting tests were carried out according to the ASTM D-6110 standard on a XJ-T0G composited impacting tester (Hebei Chengde Mechanical Instrument Co., Ltd., Chengde, China). The dimensions of the samples were 80 mm × 11 mm × 4 mm (thickness). The pendulum length was 60 mm and pendulum energy is 2.0 J with a speed of 2.9 m/s. Ten replicates for each composition were tested for impact strength. The mean value of parallel specimens was used to perform further analysis.

## 3. Results and Discussion

### 3.1. SEM Analysis

**Figure 1:**
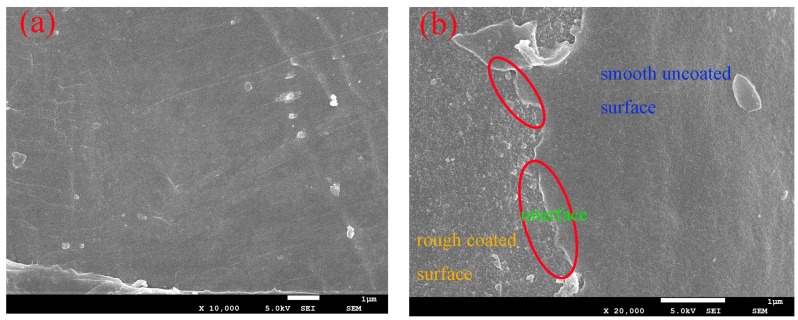
SEM images of (**a**) untreated wheat straw fiber and (**b**) wheat straw fiber treated by dopamine.

Figure 1 presents the SEM images for WSF and MMT before and after the modification. As shown in Figure 1a,b there is an obvious distinction between the unmodified WSF and dopamine-modified WSF. Raw WSF showed a continuous and smooth surface, while the dopamine-modified WSF presented a rough and uneven surface, which increased the surface area of the fiber and played an important role in enhancing the interfacial bonding strength. Meanwhile, dopamine underwent auto-oxidative polymerization in aqueous solution to formed free radicals, which then coupled to formed cross-linking bonds, formed a tightly attached polydopamine coated on the surface of WSF. The strong chemical bonding enabled the interfacial compatibility between WSF and PLA to greatly increased. The results highlighted the interface between the polydopamine and WSF. This suggests that the polydopamine layer was successfully formed on the surface of WSF and the interfacial bond strength of WSF/PLA could also be improved due to the biocompatibility of dopamine. Furthermore, the dopamine modification may also be favorable for the homogeneous dispersion of WSF in PLA.

**Figure 2:**
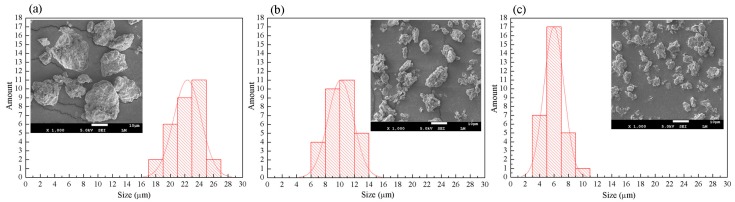
Size distribution of (**a**) Na-MMT, (**b**) ALA modified MMT and (**c**) two-step modified MMT with ALA and KH550.

Comparing Figure 2a with Figure 2b,c, it is clear that the raw MMT mainly accumulated into large aggregates with irregular surface structures, while ALA-modified MMT presented a much looser structure and smaller exfoliated separate layers. Besides, the MMT modified using ALA and KH550 demonstrated the loosest and smallest size among the three kinds of MMTs. As revealed in the size distribution chart, the untreated montmorillonite particles presented a large size distribution, while 67% of the particles possessed the size of around 23 μm. After the first treatment, the size of the particles decreased to 9–11 μm, which accounted for 70% of the total number. There was a significant size decrease in the product treated twice, while 80% of the particles had the size of 4–6 μm. The MMT sheet adsorbed exchangeable inorganic cations, while the organic cations of ALA and KH550 entered the MMT sheet by ion exchange, which enlarged the interlamellar spacing of MMT and changed its surface from hydrophilic to lipophilic, thus improved the compatibility of MMT and PLA. It is evident that the improvement in morphology can facilitate the dispersion of WSF in PLA.

### 3.2. XRD Analysis

**Figure 3:**
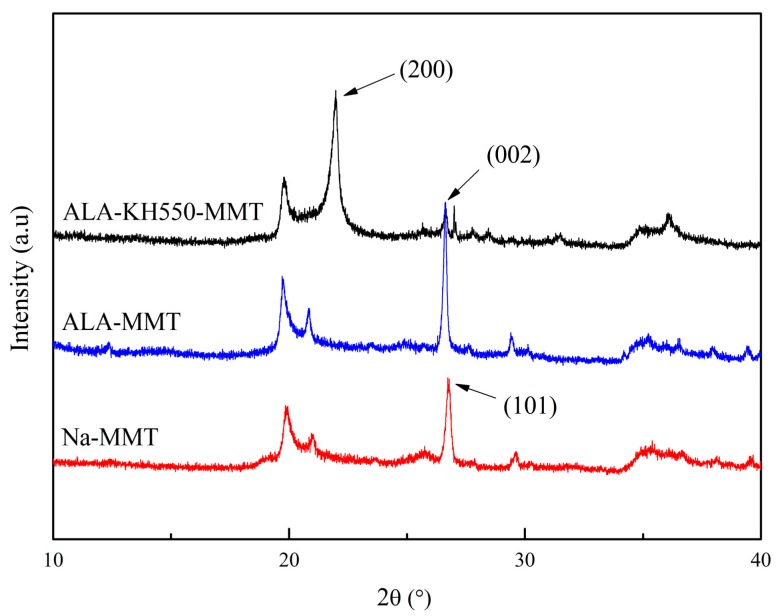
XRD patterns of Na-MMT, ALA-MMT and ALA-KH550-MMT.

**Figure 4:**
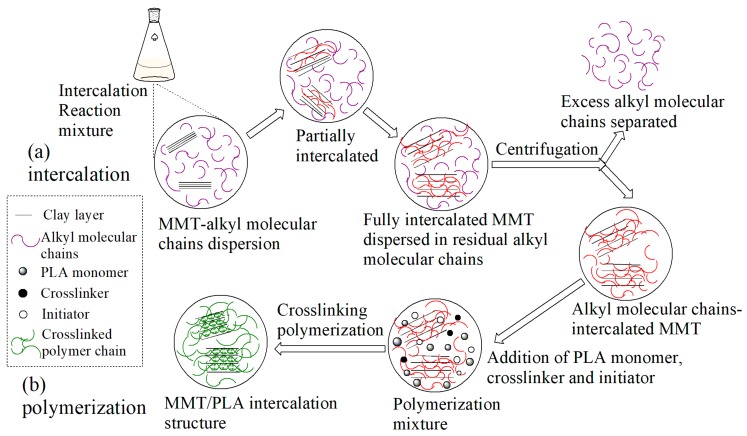
The intercalation mechanism of MMT.

Figure 3 shows the X-ray diffraction patterns of Na-MMT, ALA-MMT, and ALA-KH550-MMT. For the Na-MMT, the strong characteristic diffraction peak was at 2θ = 26.8°, which was attributed to (101) plane of MMT *I*_β_. The diffraction patterns of the ALA-MMT and ALA-KH550-MMT exhibited two typical crystalline peaks at 2*θ* = 26.6° and 22°, which corresponded to (002) semi-crystalline plane and (200) crystal plane of MMT, respectively. Additionally, the height and width of the characteristic diffraction peaks of modified MMT were higher and wider than those reported in previous publications. The diffraction peak showed a shift towards smaller angles, which indicated the increase in interlayer space and the successful intercalation of ALA molecules. The intercalation mechanism is shown in Figure 4. There were exchangeable cations between the MMT layers. Due to the hydration of interlayer cations, MMT can be suspended and dispersed in water. Through the cation exchange reaction, the organic cation can be intercalated between the MMT layers, due to which the MMT with good hydrophilicity was converted into lipophilicity, which was an important basis for realizing the nano-dispersion of MMT in the polylactic acid matrix. ALA, and KH550 were used as the organic modifier, and its alkylammonium ion entered the MMT layer through ion exchange. The surface of the sheet was covered by long carbon chain of the alkyl group, which increased the affinity of organic MMT and organic phase. At the same time, the longer alkyl molecular chains were arranged in a certain manner between the layers, which can increased the interlayer spacing and facilitated the intercalation of polylactic acid monomers into the sheets to formed a MMT/PLA intercalation structure. Compared with the ALA-MMT, the peak of ALA-KH550-MMT shifted towards further smaller angle, indicating the increase in interlayer space. The interlayer space of modified MMT was 4.06 nm, which increased by 123% compared to the unmodified MMT. This can be ascribed to the enhancement of oleophilicity by ALA molecules and the reaction activity of the interlayer. At this point, the silanol formed by the decomposition of KH550 can react with –OH on the MMT surface, which is beneficial for the intercalation of KH550 molecules into MMT layers. Therefore, it can be concluded that raw MMT was transformed into ALA-KH550-MMT after the two-step treatment process.

### 3.3. FTIR Analysis

The nature of chemical bonding between WSF and PDA was investigated using FTIR spectroscopy. The FTIR spectra of WSF and the reaction product of PDA-WSF provide spectral evidence of chemical coupling between PDA and WSF. As shown in Figure 6a the characteristic peak of ‒OH bending vibrations was at 1051 cm^−1^. The two peaks centered at about 898 and 1158 cm^−1^ were ascribed to the C‒O‒C stretching vibrations. Two absorption peaks at 2864 and 2919 cm^−1^ can be assigned to the symmetry stretching and asymmetry stretching mode of ‒CH_2_ and ‒CH_3_ groups of the HPS, respectively. The O‒H stretching vibration (hydroxyl or carboxyl) was at around 3318 cm^−1^ [26]. After the modification using PDA, the O‒H stretching vibration at around 3318 cm^−1^ shifted to lower wavenumbers, which was due to the combination of O‒H and N‒H stretching vibrations. Besides, the characteristic peaks at 2919 cm^−1^ and 2864 cm^−1^ were evidently weakened, and arose from the decrease in C‒H bond [27]. The C‒O‒C stretching vibrations at around 1086 cm^−1^ shifted to 1067 cm^−1^, which can be assigned to C‒O‒C stretching vibrations of PDA. Besides, the emergence of a new peak at 1458 cm^−1^ can be attributed to the stretching vibration of ‒C‒N from PDA. Based on the mentioned results, the successful growth of PDA into WSF surface can be confirmed.

**Figure 5:**
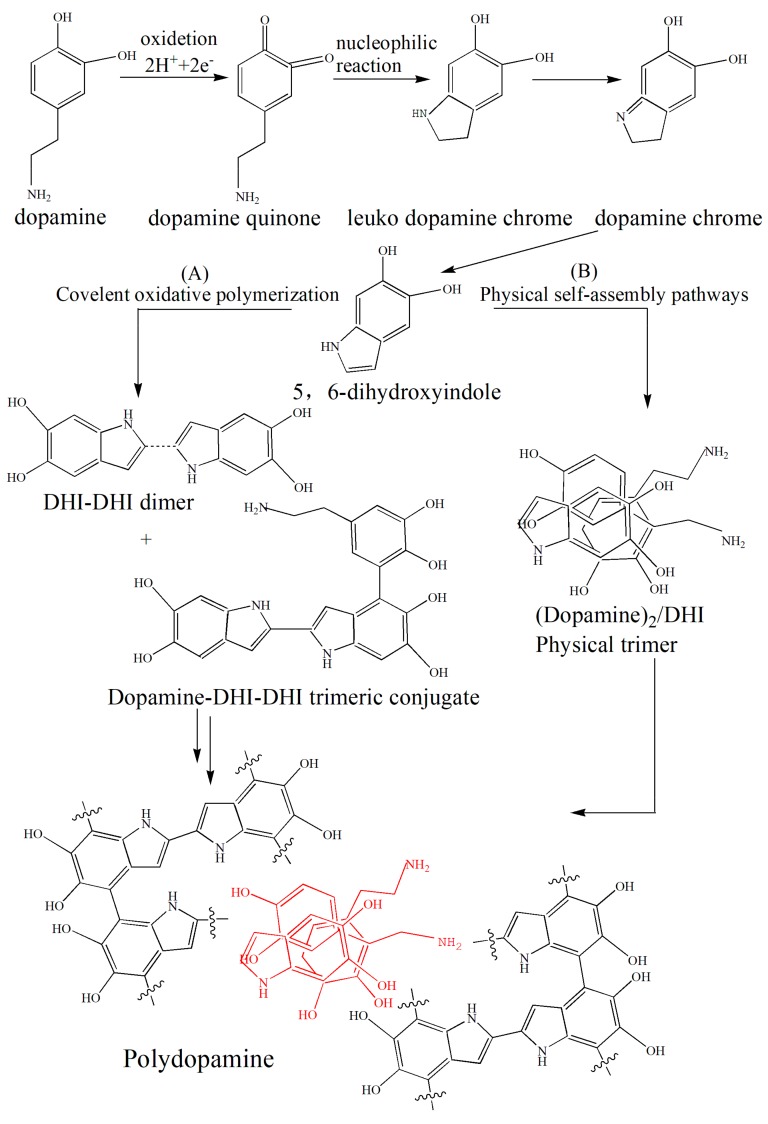
Polymerization mechanism of dopamine.

The formation mechanism of polydopamine membrane is shown in Figure 5. Under alkaline aerobic conditions, dopamine underwent oxidative self-polymerization to form polydopamine. Throughout the oxidative self-polymerization process, dopamine was oxidized to dopaminquinone by the action of alkaline environment and oxygen. Due to the instability of its molecular structure, dopamine oxime was prone to intramolecular cyclization, and formed a colorless dopamine intermediate structure. This colorless dopamine intermediate was highly susceptible to further oxidition to form a pink dopamine intermediate. The pink dopamine intermediate formed an unstable 5,6-dihydroxyindole intermediate after oxidative rearrangement. This 5,6-dihydroxyindole intermediate underwent intermolecular and intramolecular rearrangement and eventually crosslinks to formed a dark brown polydopamine coating.

**Figure 6:**
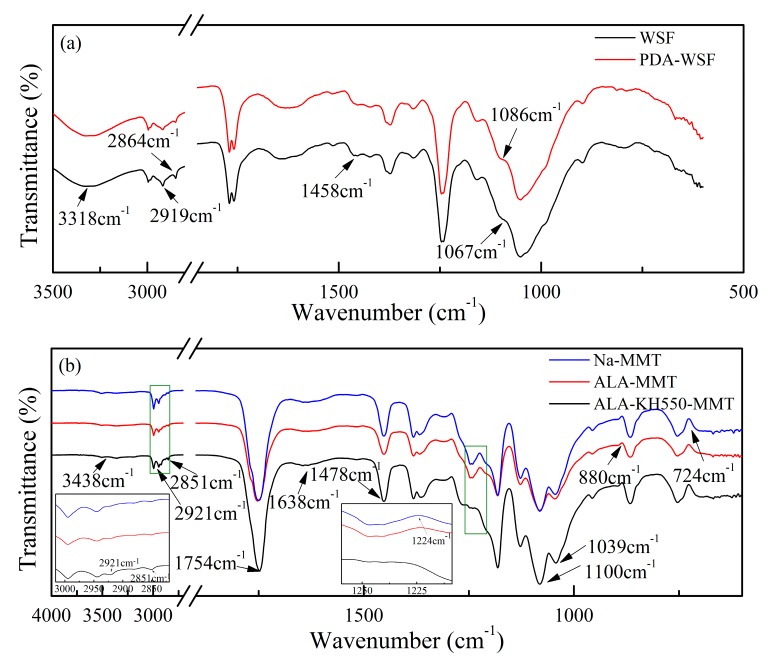
FTIR spectra of (**a**) WSF, PDA–WSF, (**b**) Na–MMT, ALA–MMT, and ALA–KH550–MMT.

The FTIR spectra of Na‒MMT, ALA‒MMT, and ALA‒KH550‒MMT are shown in Figure 6b. As for the spectrum of Na‒MMT, the stretching vibration of the absorbed water was confirmed based upon the broad absorption band at around 3438 cm^−1^. After drying, this broad absorption peak was weakened. The broad absorption band at 1638 cm^−1^ can be attributed to the bending vibration of water molecules between the MMT layer and the ‒OH groups of MMT lattice. The bending vibration of Si‒O‒Si was located at 1039 cm^−1^, while the bending vibration of MMT’s inner structure was located between 697 cm^−1^ and 914 cm^−1^. Compared with the pure Na‒MMT, the new characteristic bands of 2921 cm^−1^ and 2851 cm^−1^ were attributed to ‒CH stretching vibration, indicating that the modified MMT contained organic components, whereas the characteristic peaks appeared at 1224 cm^−1^ were ascribed to the presence of surface epoxy groups. These results demonstrated that ALA was grafted onto the MMT surface. Besides, the N‒H bending vibration in the plane was confirmed by the broad absorption band between 1560 and 1640 cm^−1^. The band at 1100 cm^−1^ originated from the vibration of Si‒O‒Si and the intensity was enhanced after the two-step treatment, which was similar to the phenomenon reported in a previous work [28], indicating the successful second modification caused by KH550. Therefore, these results support the successful two-step modification of MMT using ALA and KH550.

### 3.4. Thermogravimetric Analysis

**Figure 7:**
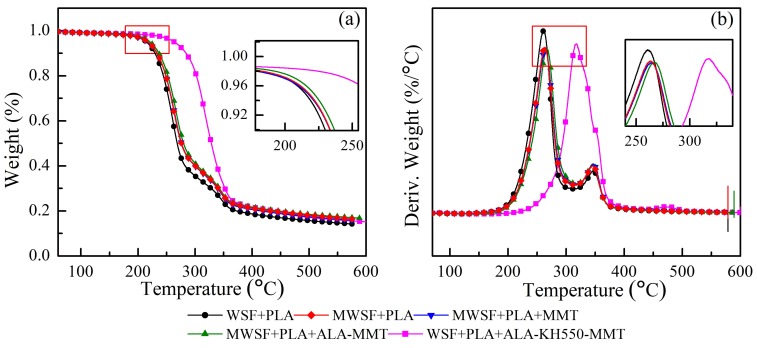
(**a**) TGA and (**b**) DTG thermograms of WSF+PLA, MWSF+PLA, MWSF+PLA+MMT, MWSF+PLA+ALA–MMT, and MWSF+PLA+ALA–KH550–MMT.

TGA and DTG techniques were used in order to estimate the weight loss kinetics of different composites. The TGA results for different samples are shown in Figure 7a. The thermal weight loss of WSF/PLA composites mainly occurred in the range of 180 °C–400 °C, and the mass loss was about 80%. This can be attributed to the reason that the composite material underwent thermal degradation at this stage, resulted in a decrease in material mass. As can be seen, the pure PLA exhibited a higher decomposition temperature (306 °C) than PLA+WSF (250 °C), indicating that the thermal stability of pure PLA decreased after the addition of WSF. According to a previous work [29], two main mass loss regions existed in the decomposition process of WSF/PLA composite under inert atmosphere. The initial mass loss with in the range of room temperature to 150 °C was attributed to the evaporation of water. The second main degradation region was located within the range of 150–400 °C, which could be ascribed to the decomposition of WSF and PLA chain. Therefore, the second mass loss was a combing process of the decomposition of WSF and PLA. The initial degradation temperature *T*_0_ and the ending degradation temperature *T_f_* were obtained from the corresponding values of the curve shown in Figure 7. It can be seen that both the *T*_0_ and *T_f_* improved after the MMT was organically modified due to the addition of ALA and ALA-KH550 to WSF/PLA composite material. This indicated that the organic treatment of MMT can improve the heat resistance of composite. Compared with the nanocomposites prepared using ALA-MMT, the *T*_0_ of ALA-KH550-MMT changed from 212 to 250 °C, whereas the *T_f_* changed from 352 °C to 366 °C. Both the temperatures of the nanocomposites prepared using ALA-KH550-MMT increased, indicated that the two-step modified MMT can effectively improved the heat resistance of PLA matrix, and the improvement effect was stronger. The two-step modified MMT layer had good barrier properties, which can hindered the thermal decomposition of products—the diffusion and volatilization of small molecules and oligomers. Furthermore, this property helped in delayed the rate of thermal degradation and improved the heat resistance of the material, which also indirectly indicated that PLA and the two-step modified MMT formed an intercalation-type nanocomposite. After the OMMT modification, the *T_d_* value decreased. This behavior may be ascribed to the OMMT surfactant ALA, which is easy to degrade under the heating effect. As the thermal degradation proceeded, the low molecular weight compounds were gradually released from the interlayers of OMMT. This process may accelerate the thermal decomposition of WSF/PLA composites, and hence, decrease the *T_d_* value.

The derivative thermogravimetric (DTG) curves of various composites are shown in Figure 7b. It can be clearly observed that the maximum rate of thermal decomposition of WSF/PLA composite was located at 260 °C, which was lower than that of the WSF/PLA modified with ALA-MMT. The main reason was that the heat flow was blocked by OMMT. This phenomenon is clear in the MMT treated by the two-step modification.

### 3.5. Study of Mechanical Properties

**Figure 8:**
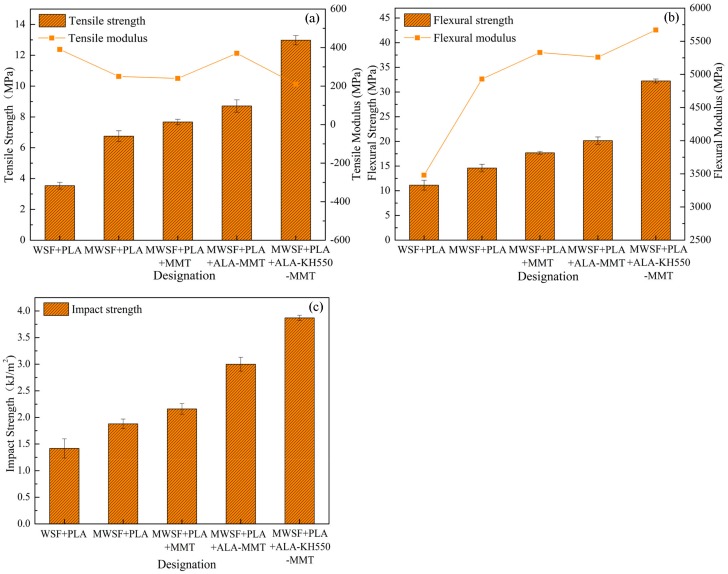
Mechanical properties of composite: (**a**) Tensile strength and tensile modulus, (**b**) Flexural strength and flexural modulus, (**c**) Impact strength.

Mechanical properties were discussed according to related articles [30], and experimental conditions and the mean value of the property was plotted in Figure 8. Tensile strength, flexural strength and the impact strength at break provide an excellent measure of the degree of reinforcement provided by the proposed modifications. It was well known that the mechanical properties of biomass composite depended on the molecular arrangement (i.e., the crystalline morphology, molecular orientation, and relaxation process of both the crystalline and amorphous regions) [31]. It can be seen from Figure 8a that the tensile strength gradually increased with the related modification method. The tensile strength was 3.54 MPa for WSF+PLA, 6.75 MPa for MWSF+PLA, 7.67 MPa for MWSF+PLA+MMT, 8.71 MPa for MWSF+PLA+ALA-MMT and 12.98 MPa for MWSF+PLA+ALA-KH550-MMT, indicating an increase of 367% compared to the unmodified MMT. This can be attributed to the addition of MMT nanoparticles with high length-to-diameter ratio and surface area, which played a significant role in the adsorption of hydroxyl groups on the surface of the fiber and chemical bonds in PLA. The miscibility of polymer blends had been studied widely [32,33,34,35], because it was important for the solid dispersion development. Here, the presence of such strong interactions between the reactive functional groups of PLA and WSF modified with polydopamine was beneficial for the good miscibility of the blend constituents, which had been confirmed by earlier report [36]. However, the tensile modulus of different samples showed a less significant change, which was due to the residual of wax coat on the surface of wheat fibers. For Na-MMT modified composites, the tensile modulus were mostly improved, which might be due to the better distribution and exfoliated structure of OMMT in PLA/WF system. When it came to the flexural strength, as shown in Figure 8b, it was 11.14 MPa for WSF+PLA, 14.6 MPa for MWSF+PLA, 17.69 MPa for MWSF+PLA+MMT, 20.16 MPa for MWSF+PLA+ALA-MMT and 32.24 MPa for MWSF+PLA+ALA-KH550-MMT. Sinha et al. [37] reported some enhancement of mechanical properties for PLA/clay composites. When the clay was well dispersed in the polymer matrix, it would lead to strong interaction between the PLA matrix and the clay, which would increase the level of intercalation. The same reason can be used to explain the better performance of OMMT than Na-MMT [38]. Flexural modulus displays a similar increasing trend for flexural strength, which has been reported in a previous work [39]. Similar trends were observed for impact strength, as shown in Figure 8c. On the one hand, the MMT/PLA intercalation structure helped to improved the stress concentration of the composite under load. On the other hand, the compatibility of MMT with the PLA matrix after KH550 treatment was improved, and a flexible interface layer was formed on the surface of fiber, which enhanced the toughness of the material. Therefore, these results show that the dopamine and the two-step modifications are beneficial for the improvement of mechanical properties.

## 4. Conclusions

In this work, an easy way to prepare a WSF/PLA composite, either treated by dopamine or by a two-step modification using MMT as a filler, was proposed. The SEM, XRD, FT-IR results demonstrated the successful modification of WSF and MMT using dopamine and ALA, respectively. OMMT showed better performance than Na–MMT due to the improvement in most of the tested properties, including the thermal decomposition temperature, flexural modulus, and flexural strength. The results of FTIR proved that the PLA matrix was successfully intercalated into OMMT, which had a positive impact on the performance of WSF/PLA composites. The TGA analysis showed that a low thermal degradation rate and high heat resistance were achieved for the WSF/PLA composite treated with modified MMT nanoparticles. Through the comparison of various samples’ mechanical properties, it was verified that both the dopamine modification of WSF and the two-step treatment exhibited a positive impact on the mechanical properties of WSF/PLA composites. This route offers researchers a potential scheme to improve the mechanical and thermal properties of PLA/WSF composites in a low-cost way.

## Figures and Tables

**Table 1:** Formulation of various samples for the preparation of the modified wheat straw fiber/PLA composites.

Code	Modified MMT (wt%)	Silane coupling agent (wt%)	Wheat straw fiber (wt%)	PLA (wt%)	Dopamine (wt%)	ZnO (wt%)	Paraffin (wt%)
A	0	2	40	54	0	1	1
B	0	2	40	54	1	1	1
C	1(Na–)	2	40	54	1	1	1
D	1(ALA–)	2	40	54	1	1	1
E	1(ALA–KH550–)	2	40	54	1	1	1
